# Influenza A plasma and serum virus antibody detection comparison in dogs using blocking enzyme-linked immunosorbent assay

**DOI:** 10.14202/vetworld.2015.580-583

**Published:** 2015-05-06

**Authors:** H. T. Lin, C. H. Hsu, H. J. Tsai, C. H. Lin, P. Y. Lo, S. L. Wang, L. C. Wang

**Affiliations:** Institute of Veterinary Clinical Sciences, School of Veterinary Medicine, National Taiwan University, 1 Sec. 4, Roosevelt Road, Taipei 10617, Taiwan

**Keywords:** dog, enzyme-linked immunosorbent assay, influenza A virus, plasma, serum

## Abstract

**Background and Aim:**

The influenza A virus (IAV) is an important zoonotic pathogen with infections also reported in dogs. IAV infections can be detected through the presence of antibodies using the enzyme-linked immunosorbent assay (ELISA). Serum is the only standard sample source; however, there is no information on the availability of other sample sources for IAV antibody detection in dogs. Compared with serum, plasma is more widely employed in most animal hospitals. The object of this study is to investigate whether plasma collected in ethylenediaminetetraacetic acid (EDTA) tubes (EDTA plasma) or heparin tubes (heparin plasma) could be used in the ELISA protocol instead of serum for IAV antibody detection in dogs.

**Materials and Methods:**

Totally, 82 matched EDTA plasma and serum sample pairs and 79 matched heparin plasma and serum sample pairs were employed using blocking enzyme-linked immunosorbent assay (bELISA). The agreement and correlation between the plasma (EDTA or heparin plasma) and serum were assessed using the agreement index kappa (kD) calculation and Pearson correlation coefficient, respectively.

**Results:**

The agreement index *k*D of EDTA plasma and serum was 1.0, and that of heparin plasma and serum was 0.85. The Pearson correlation coefficient of EDTA plasma and serum was 0.87 (p<0.01), and that of heparin plasma and serum was 0.82 (p<0.01).

**Conclusion:**

The results proved that plasma, especially EDTA plasma, could be substituted for serum in the bELISA test. This might greatly expand the clinical applicability of IAV antibody detection in dogs.

## Introduction

The influenza A virus (IAV) belongs to the *Orthomyxoviridae* family and contains at least 18 HA and 11 NA subtypes [1]. The virus has a wide host range and causes varied disease symptoms, making it an important zoonotic pathogen worldwide [2]. Interspecies transmission of an entire equine influenza A (H3N8) virus in dogs was first identified in a respiratory disease outbreak occurring in greyhounds at a racetrack in Florida in 2004 [3]. In 2007, avian-origin H3N2 canine IAV was also discovered and confirmed in South Korea [4]. Both H3N8 and H3N2 subtypes were able to cause sustained transmission among dogs [5,6]. Sporadic influenza infections in dogs were successively reported in Asia, such as H3N2, H3N1, H5N2, and H5N1 in China, South Korea, and Thailand [7-13]. The IAV infections can be detected through the antibody presence using the enzyme-linked immunosorbent assay (ELISA) [14]. Serum is the only eligible sample source for the antibody detection. Comparison studies of various sample sources for ELISA are few, except for one that used cattle serum, plasma, and bulk milk for the detection of bovine diarrhea virus antibodies [15]. Collecting plasma in ethylenediaminetetraacetic acid (EDTA) tubes (EDTA plasma) or heparin tubes (heparin plasma) is prevalent during practical use rather than serum. Whether ELISA could be employed in detecting IAV antibodies in the plasma instead of serum would affect the clinical applicability. Different samples sources for IAV antibody detection in dogs using blocking bELISA were compared in this study, including EDTA plasma, heparin plasma, and serum. This study could provide information on sample selection for the bELISA screening test in dogs.

## Materials and Methods

### Ethical approval

We did not use any animals in this study. The samples we used were redundant plasma or sera in the hospital. Therefore, the ethical approval was not applicable.

### Sample collection

Matched plasma and serum sample pairs were collected from dogs attending National Taiwan University Veterinary hospital (NTUVH) from October 2012 to February 2015, including individual EDTA plasma samples (n=82) matched with ­individual serum samples (n=82) and individual heparin plasma samples (n=79) matched with individual serum samples (n=79). The matched samples were taken from the same animal on the same day. Serum samples were collected using vacutainer serum tubes (BD, Franklin Lakes, NJ, USA). The EDTA plasma samples were collected using vacutainer EDTA tubes (BD, Franklin Lakes, NJ, USA). The heparin plasma samples were collected using vacutainer heparin plasma tubes (BD, Franklin Lakes, NJ, USA). All blood samples were centrifuged at 6,000 rpm for 10 min to separate the serum or plasma. Samples were then stored at −20°C until use.

### IAV antibody detection

All samples were tested for IAV antibodies using a species-independent, bELISA IAV Antibody Test Kit (IDEXX, Westbrook, ME, USA). Antibodies in samples against IAV were detected through the decrease in optical density (OD) by blocking the anti-influenza A nucleoprotein monoclonal antibody which was provided in the kit. The procedure was performed according to the manual and is briefly described below. 15 *m*L of the sample was diluted in 135 *m*L of dilution buffer. 100 *m*L of each diluted sample, undiluted negative and positive controls were dispensed into an antigen plate. This plate was incubated at 18-26°C for 60 min and then washed with washing solution. A 100 *m*L of anti-influenza A conjugate was added into each well and incubated for 30 min. The plate was washed again and 100 *m*L of TMB substrate solution was dispensed into each well. After 15 min, 100 *m*L of stop solution was added to stop the reaction. The OD of samples and controls was measured at 650 nm using an ELISA reader (TECAN, Seestrasse, Switzerland). The OD value of the sample was divided by the OD value of the negative control to obtain the S/N ratio. If the S/N ratio is <0.60, the sample is interpreted as IAV antibody positive. Conversely, if S/N ratio is ≥0.60, it is considered as IAV antibody negative.

### Sensitivity, specificity, and statistical analysis

The sensitivity and specificity of the bELISA for plasma samples were determined using serum as the gold standard. Based on the formulas in which the specificity is equal to the true negatives/(true negatives + false positives) and the sensitivity is equal to the true positives/(true positives + false negatives), the specificity and sensitivity of the bELISA using EDTA plasma or heparin plasma were calculated. The agreement and correlation between the plasma (EDTA or heparin plasma) and serum were assessed using agreement index kappa (*k*D) calculation and the Pearson correlation coefficient, respectively.

## Results

**Figure-1:**
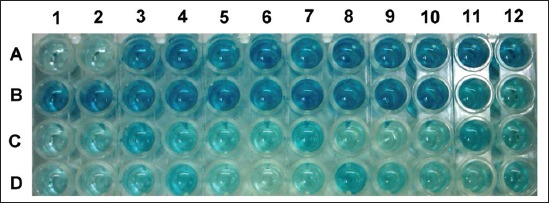
Partial sample results on the blocking enzyme-linked immunosorbent assay plate. A1 and A2: Positive control (double repeat). A3 and A4: Negative control (double repeat). A5-12 and B1-10: Serum samples, S/N≥0.6 (negative). C11: Ethylenediaminetetraacetic acid (EDTA) plasma samples, S/N≥0.6 (negative). B11: Heparin samples, S/N≥0.6 (negative). D4-D12: Serum samples, S/N<0.6 (positive). C7-10, C12, and D1-3: EDTA plasma samples, S/N<0.6 (positive). B12 and C1-6: Heparin plasma samples, S/N<0.6 (positive).

Partial sample results on the bELISA plate were shown in Figure 1. Eight EDTA plasma samples and their 8 matched serum samples showed positive among the 82 matched sample pairs (Table-1). Six heparin plasma samples and their 6 matched serum samples also showed positive. Two heparin plasma samples, however, showed negative, but their matched sera samples were positive among the 79 matched sample pairs (Table-2). Relative to the serum sample bELISA results, both the specificity and sensitivity of the bELISA using EDTA plasma as the samples were 100%. Comparably, the specificity and sensitivity of the bELISA using heparin plasma as samples were 100% and 75%, respectively.

**Table 1:** ELISA test results of 82 individually matched serum/EDTA plasma samples.

	bELISA (serum)	

	+	-
bELISA (EDTA plasma)		
+	8	0
-	0	74

bELISA=Blocking enzyme-linked immunosorbent assay, EDTA=Ethylenediaminetetraacetic acid

**Table 2:** ELISA test results of 79 individually matched serum/heparin plasma samples.

	bELISA (serum)	

	+	-
bELISA (heparin plasma)		
+	6	0
-	2	71

bELISA=Blocking enzyme-linked immunosorbent assay

The bELISA reliability between EDTA plasma and serum was calculated using the *k*D index and revealed the value was 1.0, showing perfect agreement between these two kinds of samples. The *k*D index value between heparin plasma and serum was 0.85, indicating almost perfect agreement between the two kinds of samples. Highly significant Pearson’s S/N ratio correlation was also found between EDTA plasma and serum (r=0.87, p<0.01) as well as that between heparin plasma and serum (r=0.82, p<0.01).

Although the bELISA kit manual indicated that serum is the only sample source for the test, the study results showed that either EDTA plasma or heparin plasma was feasible for IAV antibody detection. This may greatly expand its clinical usage.

## Discussion

The serological results from this study indicated the presence of IAV infection in dogs in Taiwan. Further work using large-scale seroprevalence investigation, virus isolation, and subtyping would be necessary.

The coating antigen on the bELISA plate was nucleoprotein in this study, which is highly conserved among all IAV subtypes, making this test application feasible for an extensive number of animal species beyond birds [16]. The kit manual instructs that serum is the sole recommended sample source. However, serum is collected less routinely than EDTA plasma and heparin plasma in many animal hospitals. Our study showed that EDTA plasma and heparin plasma were both good sample sources besides serum for the IAV bELISA test in dogs. Although the heparin plasma indicated lower *k*D and Pearson’s correlation coefficient than EDTA plasma matched with the serum, it was still highly usable. There were two disagreement matched sample pairs which showed S/N values near to the threshold value of 0.6. One pair displayed that the heparin plasma was 0.677 (IAV negative) and the serum was 0.575 (IAV positive), and the other pair showed that the heparin plasma was 0.648 (IAV negative) and the serum was 0.589 (IAV positive). Whether this disagreement may occur when the detected values fall around the threshold is worth further investigation.

NTUVH routinely employs EDTA-anticoagulated blood and heparin plasma for hematological and chemistry evaluation, respectively. Serum is seldom used except for certain specific items. This is the reason why only a small number of matched sample pairs were collected over a long time. It would be much better if more matched sample pairs could be collected to make the statistical data more reliable.

## Conclusion

Serum is the only eligible bELISA sample source for the detection of IAV antibodies in dogs. No information on the applicability of EDTA plasma or heparin plasma is available, which are more commonly used in many animal hospitals. The results from this study proved that plasma, especially EDTA plasma, could be used as a substitute for serum for IAV antibody detection in dogs. This could greatly assist in IAV diagnosis.

## Authors’ Contributions

LCW designed the study and drafted the manuscript. HTL and CHH carried out the study. HJT, CHL, PYL, and SLW participated in the scientific discussion. All authors read and approved the final manuscript.
